# Disentangling Membrane Dynamics and Cell Migration; Differential Influences of F-actin and Cell-Matrix Adhesions

**DOI:** 10.1371/journal.pone.0135204

**Published:** 2015-08-06

**Authors:** Jacob M. Kowalewski, Hamdah Shafqat-Abbasi, Mehrdad Jafari-Mamaghani, Bereket Endrias Ganebo, Xiaowei Gong, Staffan Strömblad, John G. Lock

**Affiliations:** 1 Karolinska Institutet, Department of Biosciences and Nutrition, Huddinge, Sweden; 2 Division of Mathematical Statistics, Department of Mathematics, Stockholm University, Stockholm, Sweden; Seoul National University, Republic of Korea

## Abstract

Cell migration is heavily interconnected with plasma membrane protrusion and retraction (collectively termed “membrane dynamics”). This makes it difficult to distinguish regulatory mechanisms that differentially influence migration and membrane dynamics. Yet such distinctions may be valuable given evidence that cancer cell invasion in 3D may be better predicted by 2D membrane dynamics than by 2D cell migration, implying a degree of functional independence between these processes. Here, we applied multi-scale single cell imaging and a systematic statistical approach to disentangle regulatory associations underlying either migration or membrane dynamics. This revealed preferential correlations between membrane dynamics and F-actin features, contrasting with an enrichment of links between cell migration and adhesion complex properties. These correlative linkages were often non-linear and therefore context-dependent, strengthening or weakening with spontaneous heterogeneity in cell behavior. More broadly, we observed that slow moving cells tend to increase in area, while fast moving cells tend to shrink, and that the size of dynamic membrane domains is independent of cell area. Overall, we define macromolecular features preferentially associated with either cell migration or membrane dynamics, enabling more specific interrogation and targeting of these processes in future.

## Introduction

Cell migration is a fundamental biological process, involved in both physiological phenomena, such as morphogenesis, and pathophysiological conditions, such as cancer metastasis. Several types of single cell migration have been described, yet these are most commonly divided into amoeboid and mesenchymal modalities [1]. The mesenchymal mode of cell migration requires the formation of protrusions at the cell’s leading edge, while trailing edges must retract, enabling cell translocation through the coordination of these so-called “membrane dynamics” [2, 3]. As such, the complex relationships between membrane dynamics, associated cell shape changes and cell migration have been extensively examined [4–7]. Resulting empirical observations and modeling have indicated strong correlative links between membrane dynamics and cell migration, an outcome that is entirely intuitive and expected. However, in a recent study comparing the respective dependence of membrane dynamics and cell migration on growth factors, Meyer *et al*. [8] made the unexpected finding that membrane dynamics in 2D more strongly predicted cell migration capacity in 3D environments than did 2D cell migration. This implies that despite being overlapping processes, membrane dynamics and cell migration are at least partially independent. Accordingly, establishing a method to disentangle the regulatory dependencies of these processes may pave the way for a more precise understanding of cell motility as a whole, where these coupled yet independent processes can be deconvolved.

The molecular underpinnings of both membrane dynamics and cell migration are immensely complex. Yet two macromolecular machineries in particular, the filamentous (F)-actin cytoskeleton and Cell-Matrix Adhesion Complexes (CMACs), play major roles in both processes [9]. Specifically, the F-actin cytoskeleton develops both the protrusive and contractile forces required for membrane extension, retraction and cell movement [10–14], while CMACs attach cells to the extracellular matrix (ECM), enabling the application of cytoskeleton-derived forces. Integrins, which bind directly to ECM ligands via their extracellular domains, form the core of CMACs. Upon activation and clustering, integrins establish large macromolecular signaling and F-actin-adaptor complexes through direct and indirect cytoplasmic tail interactions, thus forming a mechanical bridge between the ECM and F-actin cytoskeleton [15, 16]. When mature, CMACs contain hundreds of proteins [17–20], amongst which paxillin–used herein as a CMAC marker–is a central and near omni-present component. Importantly, paxillin associates closely with proteins such as vinculin that connect CMACs and F-actin [21], with mechanotransduction through this link significantly shaping CMAC dynamics [22]. Due to their pivotal roles, analyses of both F-actin and CMAC machineries through quantitative imaging can provide a rich sampling of the state of the broader cell migration system [23–25]. Such a quantitative sampling may provide a sound basis for the statistical disentangling of the overlapping processes of cell migration and membrane dynamics. Notably, using quantitative multi-scale imaging to simultaneously assess the organization of critical machineries (F-actin, CMACs; macromolecular scale) and related cell processes (membrane dynamics, migration; cellular scale), it is possible to leverage natural cellular heterogeneity (between cells and over time) to define selective dependencies [23, 26]. Such a statistical approach provides an alternative to the use of disruptive perturbations for the inference of functional associations. This is advantageous given that it is extremely difficult to experimentally target one process but not the other, while more broadly, the systemic consequences of most molecular perturbations are both extensive and unpredictable [27].

**Fig 1:**
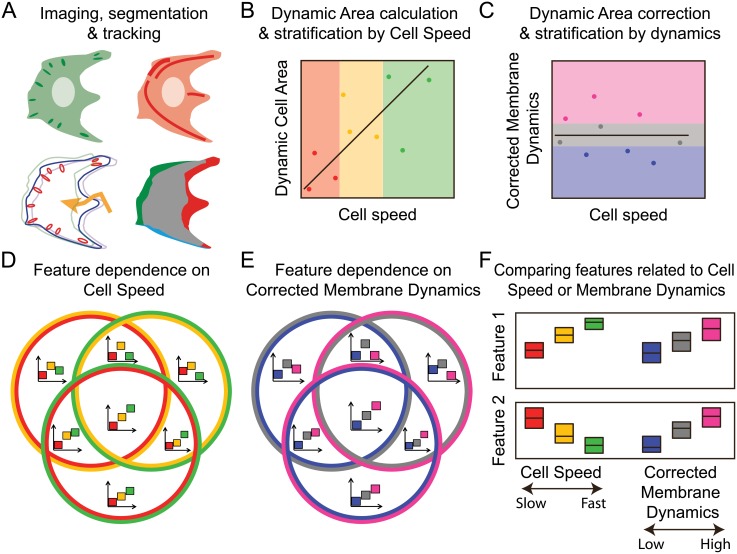
Methodological overview. (A) Schematic of live H1299 cells expressing EGFP-paxillin (Green, upper left) and RubyRed-LifeAct (red, upper right) that were imaged at 5 min intervals for 8 h. (Lower left) Segmentation identified the cell border (blue) and Cell-Matrix Adhesion Complexes (CMACs) (red). These were tracked over time, allowing extraction of static and dynamic *features* describing cell, CMAC and F-actin characteristics per cell, per time point. (Lower right) Consecutive frames were compared and protrusive (green), retractive (red), short-lived (blue) and stable (gray) regions were identified. The identification of these regions allowed for per cell quantification of both *processes* of interest, membrane dynamics and cell migration. (B) Dynamic Cell Area, defined as the total non-stable area (total area of protrusions, retractions and short-lived regions), is linearly dependent on Cell Speed. Observations were stratified into equally sized Cell Speed groups: slow (red); moderate (yellow); or fast (green). (C) To establish a Cell Speed-independent measure of membrane activity, Corrected Membrane Dynamics (CMD) was calculated by subtracting the linear dependence between Cell Speed and Dynamic Cell Area. CMD data was also stratified into equal groups with: low (blue); intermediate (gray), or; high (pink) activity. (D-E) Venn diagrams summarize the frequencies of particular relationship structures between features and changing values of Cell Speed (D) or CMD (E). Each circle of the Venn diagrams contain two colors (as defined in B-C), indicating the pairs of Cell Speed or CMD groups between which feature values were statistically compared, e.g. slow (red) vs moderate (yellow) migrating cells in D. Segments of the Venn diagram indicate which combinations of these pairwise statistical comparisons revealed significant differences in feature values. To aid interpretation, schematic archetypes (small graphical insets) are included to indicate the generalized relationship structure that is observed between each feature (Y-axes) and either Cell Speed or CMD (X-axes). Where boxes do not overlap in the Y-axis, statistically significant differences were detected between feature values in the corresponding Cell Speed or CMD groups. For example, features associated with the category represented in the lower segment of D would have significantly different values when comparing cells migrating slow (red) *versus* fast (green), but not between slow and moderate, or moderate and fast migrating cells. Hence, change in the values of such features would proceed slowly but progressively over the full range of observed Cell Speeds. Note that the actual sign of feature responses may be inverted compared to these generalized archetypes. (F) Finally, Cell Speed- and CMD-related features were identified and compared using a stringent approach. This resulted in lists of features that are related to Cell Speed, to CMD or to both processes. Of those features related to both processes, some showed equivalent responses (feature 1 example), while others showed distinct and even opposite dependencies to each process (feature 2 example).

Following the rationale outlined above (and the experimental and analytical approach summarized in Fig 1), we here infer selective correspondences between core macromolecular (CMAC and F-actin)- and cell morphology-features on the one hand, and either cell migration or membrane dynamics on the other. This is based on imaging and quantitative multi-scale analysis of single migrating cells expressing markers for F-actin and CMACs (Fig 1A). To define and disentangle properties correlating with either migration or membrane dynamics, we first determined the relationship between the proportion of cell area involved in membrane dynamics (Dynamic Cell Area) and Cell Speed (Fig 1B). The linear dependence identified was subsequently corrected for, establishing Corrected Membrane Dynamics (CMD) as a novel Cell Speed-independent measure of the degree of membrane dynamics (Fig 1C). We next characterized the structure of correlative dependencies between recorded macromolecular or cellular features and the two biological processes of interest. This was achieved by comparing cell observations stratified by Cell Speed (Fig 1D) or CMD (Fig 1E) values. Finally, we identified features that were differentially related to Cell Speed or CMD (Fig 1F), as well as whether features linked to both processes showed correlated or anti-correlated responses.

Broadly, we find that CMAC properties are more strongly related to cell migration, and that F-actin properties are more closely linked to membrane dynamics. Several features, including paxillin levels per CMAC (associated with the CMAC-F-actin-bridging adaptor complex), correlate strongly with both processes. In addition, we detail the structure of these relationships, including mapping non-linear and context-dependent correspondences. We thus utilize natural cellular heterogeneity to identify and characterize preferential associations underlying two highly interconnected biological processes. Overall, this study provides novel insights into cell migration biology, as well as a generalizable strategy for the perturbation-independent assessment of highly interdependent cell biological processes.

## Results

### Quantitative imaging and feature extraction

**Fig 2:**
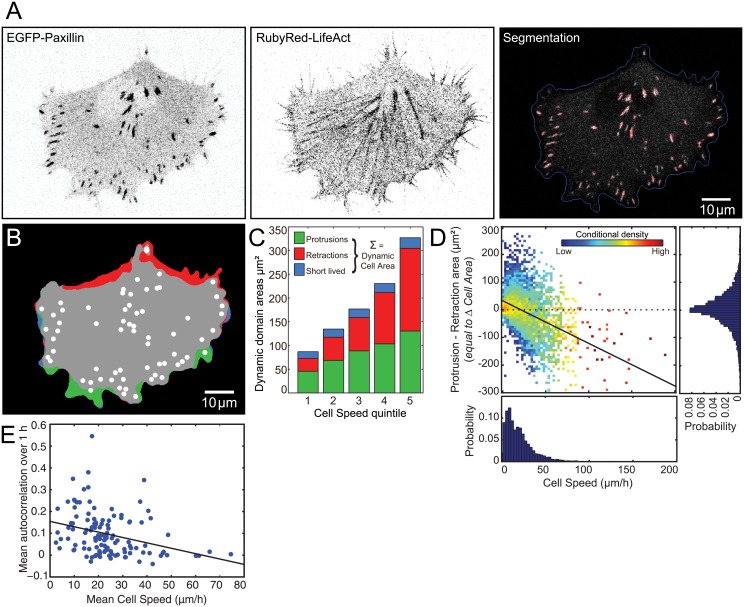
Quantitative imaging and analysis reveals that slow migrating cells increase in area while fast migrating cells decrease in area. (A) Live H1299 P/L cells expressing EGFP-paxillin and RubyRed-LifeAct were imaged and segmented in order to identify individual cells and their cohort of Cell-Matrix Adhesion Complexes (CMACs). EGFP-paxillin and RubyRed-LifeAct channels are displayed in inverted gray scale (high intensity is black). The segmentation image shows the EGFP-paxillin channel with the cell border identified in blue, CMAC borders in red and CMAC major axes in cyan. Scale bar: 10 μm. (B) By comparing consecutive frames, protrusions (green), retractions (red), short-lived (blue) and stable (gray) regions were identified, as described in Materials and Methods. White circles indicate the locations of CMACs. (C) The average size of each type of dynamic cell region (per frame) in the dataset was calculated and stratified per quintile of Cell Speed. The total height of each bar (the sum of protrusion, retraction and short-lived areas per frame) corresponds to the Dynamic Cell Area. We observed that this quantity increases with Cell Speed. (D) Scatter Plot: The net value of protrusion minus retraction areas (delta (Δ) Cell Area, μm^2^) is shown as a function of Cell Speed. The density of observations at a given Cell Speed (Cell Speed conditional density) is color-coded following log transformation, enabling better observation of trends in Δ Cell Area values given changing Cell Speed. A linear fit (Pearson’s correlation coefficient *r* = -0.27, *P* = 4.39·10^−233^) of the relationship is indicated (black line). Probability distributions: of Cell Speed (X axis), and; Δ Cell Area (Y axis, right) show a heavy-tailed distribution of Cell Speed where most cells are slow moving and few are fast moving, while Cell Area changes are approximately symmetrical overall. Notice, however, that the relatively few fast moving cells are decreasing in area substantially (retraction area is much larger than protrusion area), while the numerous slow moving cells only grow slightly, on average. (E) Mean Cell Speed autocorrelation coefficients (Y axis) are plotted conditioned upon the mean Cell Speed (X axis) of each cell. Autocorrelation values were calculated per cell trajectory with a maximum time lag of 1 h (12 frames). A linear fit (Pearson’s correlation coefficient *r* = -0.32, *P* = 0.00037) of the relationship indicates that autocorrelation of Cell Speed is lower in cells with higher average Cell Speeds. This indicates that the temporal persistence of Cell Speed correlates negatively to Cell Speed itself. See also S1 Movie, showing the same cell as in A-B.

Live H1299 human non-small lung cancer cells, stably transfected with EGFP-paxillin, a core Cell-Matrix Adhesion Complex (CMAC) component [28], and RubyRed-LifeAct, an F-actin marker [13, 29] (referred to here as H1299 P/L cells [23]), were imaged via confocal microscopy during random migration. Images were segmented to identify individual cells and their CMAC cohort (Fig 2A), and then tracked to capture cell and CMAC dynamics (see Materials and Methods, [23]). A spectrum of quantitative features were extracted defining the morphology and dynamics of individual cells as well as their CMACs and F-actin. These 150 parameters (summarized in S1 Table) provide a partial characterization of the cellular state. Overall, following data parsing (Materials and Methods), the analyzed data set contained 6419 cell observations, incorporating 177938 CMAC observations, derived from 122 cells over 19 independent experiments. Crucially, membrane dynamics were characterized by comparing segmented cell boundaries over three consecutive time points, defining membrane protrusions, membrane retractions and short-lived regions (stable for one time point only) (Fig 2B and S1 Movie). We defined the Dynamic Cell Area as the sum of the areas of protrusions, retractions and short-lived regions, per cell, per time point.

### Slowly migrating cells increase in area while fast migrating cells decrease in area

We next divided the data set comprising all cells into quintiles of Cell Speed. The total per cell area of protrusions, retractions and short-lived regions was calculated and the average results per Cell Speed quintile are displayed (Fig 2C). Note that the sum of these values equates to the total Dynamic Cell Area, which increases strongly with rising Cell Speed. This indicates a positive correlation between Cell Speed and Dynamic Cell Area, as expected.

It is noteworthy that the balance between protrusion and retraction is unequal at most speeds, with slow moving cells having proportionally more area associated with protrusion (Fig 2C, quintile 1), and fast moving cells having proportionally more area associated with retraction (Fig 2C, quintile 5). A continuous analysis of this imbalance, where retractive area is subtracted from protrusive area to reveal net changes in area per cell observation (Fig 2D, scatter plot), confirms that slow moving cells tend to be growing, whereas fast moving cells tend to be shrinking. More specifically, we see that while the overall distribution of changes in Cell Area (protrusion minus retraction area histogram, Fig 2D) is virtually symmetrical, the majority of cells are both slow moving (Cell Speed histogram, Fig 2D) and growing slightly, while fast moving cells shrink rapidly but are relatively uncommon. Thus, the Cell Area distribution is equilibrated by numerous small area increases (slow cells) on the one hand, and infrequent large area decreases (fast cells) on the other. Interestingly, this strong correspondence between Cell Speed and changes in Cell Area implies that cells must regularly change speed to maintain their size. In particular, rapid migration, when cells shrink dramatically, should be especially transient. This hypothesis was confirmed by an analysis of Cell Speed autocorrelation (Fig 2E), where cells with high average speeds show limited autocorrelation (unable to sustain persistently fast motility) in comparison to cells with low average speeds (relatively consistent slow motility).

Importantly, Dynamic Cell Area is a size-independent indicator of membrane dynamics, since no dependence was detected between Cell Area and Dynamic Cell Area (S1A Fig). This means that the proportion of cell area that is dynamic tends to decrease as Cell Area increases (S1B Fig). Overall, this implies that cells at a given speed tend to organize a characteristic amount of their membrane (and underlying protrusive and contractile machinery) into dynamic domains, rather than that these domains scale with cell size.

### Corrected Membrane Dynamics: a Cell Speed-independent measure of relative membrane dynamics

**Fig 3:**
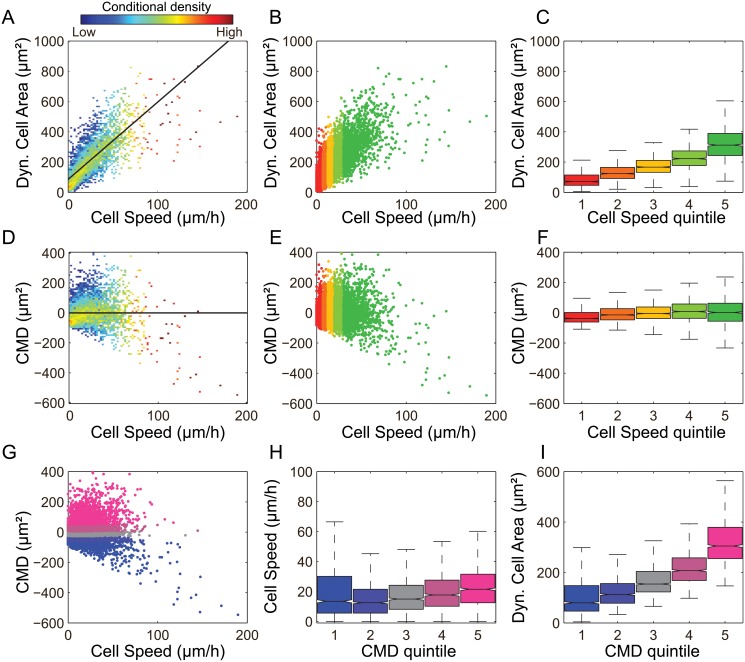
Speed and Corrected Membrane Dynamics are independent. (A) Values for Cell Speed and Dynamic Cell Area are plotted. The density of observations at a given Cell Speed (Cell Speed conditional density) is color-coded following log transformation, enabling better observation of trends in Dynamic Cell Area values given changing Cell Speed. A linear fit (Pearson’s correlation coefficient *r* = 0.74) of the relationship between Cell Speed and Dynamic Cell Area is indicated (black line). (B) Scatter plot of Cell Speed versus Dynamic Cell Area. Cell observations are divided into Cell Speed quintiles as indicated by color scaling: red (slow, 0%-20%); orange (20%-40%); yellow (moderate, 40%-60%); light-green (60%-80%); green (fast, 80%-100%). (C) Box plots show the median and variability of Dynamic Cell Area per speed quintile. The box shows the quartiles and the whiskers show 1.5 times the interquartile range (IQR). Outliers are not shown. Notches are placed at the median value $\pm 1.57\text{ IQR}/\sqrt{n}$, where *n* is the number of observations in each quintile (approximation of the 95% confidence interval of the median). By comparing these measures for each Cell Speed quintile we observed a monotonic increase of Dynamic Cell Area with Cell Speed. Colors as in (B). (D) Corrected Membrane Dynamics (CMD) is defined by subtracting the linear relationship between Cell Speed and Dynamic Cell Area from all observations. Conditional density color-coding and linear fit are calculated and displayed as in (A). (E) Scatter plot of Cell Speed versus CMD, color-coded by Cell Speed quintiles as in B. (F) Box plots as in (C) based on Cell Speed quintiles show that there is no trend in CMD as a function of Cell Speed. Box plots structured as in C. Colors as in (B). (G) Scatter plot of Cell Speed versus CMD. The observations were divided into equally sized quintiles of CMD as indicated by color scaling: blue (low, 0%-20%); purple (20%-40%); grey (intermediate, 40%-60%); dark-pink (60%-80%); pink (high, 80%-100%). (H) Box plots as in (C) showing median and variability of Cell Speed per CMD quintile. Colors as in (G). (I) Box plots as in (C) showing median and variability of Dynamic Cell Area per CMD quintile. Colors as in (G).

As noted in Fig 2C, Cell Speed and Dynamic Cell Area are positively correlated. To better define this correlation, we plotted values for each cell observation, revealing an approximately linear dependence between Cell Speed and Dynamic Cell Area (Fig 3A–3C). To compare the relative level, or degree, of membrane dynamics for each cell independently of Cell Speed, we then corrected for this linear dependence. Specifically, the linear fit between Cell Speed and Dynamic Cell Area was subtracted from Dynamic Cell Area. This defined a new, Cell Speed-independent measure: Corrected Membrane Dynamics (CMD). Plotting CMD against Cell Speed confirms the independence of these parameters (Fig 3D–3F). Similarly, by dividing the dataset into quintiles of CMD, we observe again that CMD is almost entirely independent of Cell Speed (Fig 3G and 3H), while a strong dependency exists between CMD and Dynamic Cell Area, as expected (Fig 3I).

### Strategy for analysis of relationship structures

**Fig 4:**
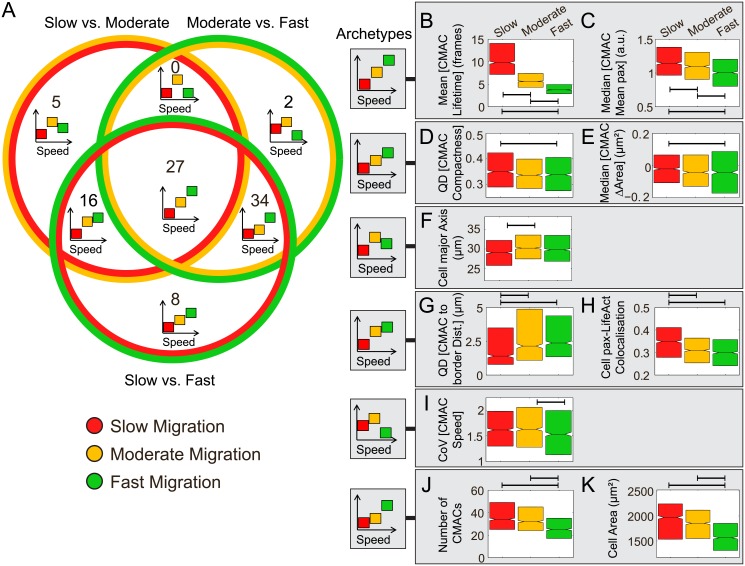
Analysis of relationship structures between features and Cell Speed. (A) A Venn diagram summarizes the frequencies of particular relationship structures between features and changing values of Cell Speed. Each circle of the Venn diagram contains two colors, indicating the Cell Speed quintiles (red, slow; yellow, moderate; green, fast) between which feature values were compared via pairwise testing (slow *versus* moderate; moderate *versus* fast; slow *versus* fast). Segments of the Venn diagram indicate which combinations of pairwise tests (Wilcoxon rank sum test with Bonferroni correction) resulted in statistically discernable differences. The number of features is indicated for which a given combination of tests showed significance. To aid interpretation, schematic archetypes are included to indicate the type of correspondence that is observed between each feature and Cell Speed. Where boxes do not overlap in the Y-axis, statistically significant differences were detected between feature values in the corresponding Cell Speed groups. Note that the actual sign of feature responses may be inverted compared to these generalized archetypes. (B-K) The observed archetypes from (A) are illustrated to the left and examples of features corresponding to each archetype are shown in boxes to the right. Comparison brackets in each panel indicate significant differences (P<0.001 after Bonferroni correction, see Materials and Methods). Box plots show quartiles. Outliers are not shown. Notches are placed at the median value $\pm 1.57\text{ IQR}/\sqrt{n}$, where *n* is the number of observations in each quintile (approximation of the 95% confidence interval of the median). (B) Mean Cell-Matrix Adhesion Complex (CMAC) Lifetime and (C) median of CMAC Mean paxillin intensity per cell both show stably monotonic decreases across all Cell Speeds. (D) Quartile dispersion (QD) of CMAC compactness is significantly lower in fast than in slow cells. (E) The median rate of change in CMAC area is negative meaning that CMACs are shrinking. This shrinking is more rapid in fast than in slow cells. (F) Cell major Axis is significantly higher in moderate than in slow cell observations, but not between any other groups. (G) QD of CMAC to cell border distance at each time point increases significantly between slow and moderate but not between moderate and fast cells; (H) paxillin-actin colocalization on a cell level decreases in a corresponding way. (I) Coefficient of variation (CoV) of CMAC Speed is significantly lower in fast than in moderate cell observations. (J) The number of CMACs per cell at each time point and (K) the total area of the cell at each time point both show a monotonic decrease between moderate and fast cells.

**Fig 5:**
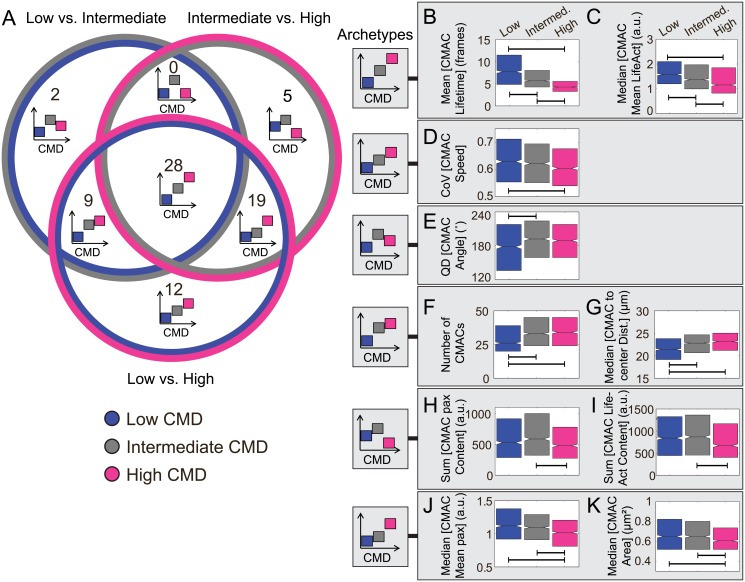
Analysis of relationship structures between features and Corrected Membrane Dynamics. (A) A Venn diagram summarizes the frequencies of particular relationship structures between features and changing values of Corrected Membrane Dynamics (CMD). Each circle of the Venn diagram contains two colors, indicating the CMD quintiles (blue, low; grey, intermediate; pink, high) from which feature values were compared via pairwise testing (low *versus* intermediate; intermediate *versus* high; low *versus* high). Segments of the Venn diagram indicate which combinations of pairwise tests (Wilcoxon rank sum test with Bonferroni correction) resulted in statistically discernable differences. The number of features is indicated for which a given combination of tests showed significance. To aid interpretation, schematic archetypes are included to indicate the type of correspondence that is observed between each feature and CMD. Where boxes do not overlap in the Y-axis, statistically significant differences were detected between feature values in the corresponding CMD groups. Note that the actual sign of feature responses may be inverted compared to these generalized archetypes. (B-K) The observed archetypes from (A) are illustrated to the left and examples of features corresponding to each archetype are shown in boxes to the right. Comparison brackets in each panel indicate significant differences (P<0.001 after Bonferroni correction, see Materials and Methods). Box plots show quartiles. Outliers are not shown. Notches are placed at the median value $\pm 1.57\text{ IQR}/\sqrt{n}$, where *n* is the number of observations in each quintile (approximation of the 95% confidence interval of the median). (B) Mean CMAC lifetime and (C) median of Cell-Matrix Adhesion Complex (CMAC) mean LifeAct intensity per cell both show stably monotonic decreases across all CMD groups. (D) Coefficient of variation (CoV) of CMAC Speed is significantly different only between cell observations with low and high CMD, but not other pairs of groups. (E) Quartile dispersion (QD) of CMAC angle indicates the CMAC angle variability within a cell is significantly different between low and intermediate cell observations only. (F) The number of CMACs and the (G) median CMAC to center distance at each point in time both show a significant increase between the low and moderate CMD groups. The sum of paxillin (H) or LifeAct (I) content in the Cell-Matrix Adhesion Complexes (CMACs) both show a significant decrease between the intermediate and high CMD groups. They both indicate a possible maximum value at intermediate level. (J) Median of the CMAC paxillin intensity and (K) median CMAC Area both decrease significantly between intermediate and high CMD groups.

The establishment of independent measures for cell migration (Cell Speed) and membrane dynamics (CMD) enabled analysis of correspondences between either *process* and each of the 150 *features* defining underlying cell, CMAC and F-actin organization and dynamics. To define the structure of these feature-process relationships, we designed our analysis to provide high sensitivity to both non-linear and non-monotonic trends, i.e. where relationships are contextually dependent on different levels of Cell Speed and/or CMD. This was achieved through quintile-based stratification of cell observations according to Cell Speed (as in Fig 3B) or CMD (as in Fig 3G). We then selected observations in quintiles 1 (0–20), 3 (40–60%) and 5 (80–100%) of either Cell Speed (designated “slow”, “moderate” or “fast”, respectively) or CMD (designated “low”, “intermediate” or “high”, respectively). For each of the 150 features assessed, the Wilcoxon rank sum test (with Bonferroni correction) was applied to determine if significant differences existed between feature values in quintiles 1 *versus* 3, 3 *versus* 5, and 1 *versus* 5 (see Materials and Methods). Testing outcomes for all 150 features are displayed in S1 Table. By identifying exactly where feature values diverged, we comprehensively characterized the structure of relationships between each feature and Cell Speed (summarized in Fig 4) and/or Corrected Membrane Dynamics (summarized in Fig 5), in terms of direction, linearity and monotonicity.

### Feature relationships to Cell Speed are frequently non-linear and context-dependent

A Venn diagram encapsulates the results of the inter-quintile testing regime for Cell Speed, described above (i.e. Slow vs Moderate, Moderate vs Fast, Slow vs Fast, Fig 4A). Each segment of the diagram indicates which combination of the three statistical tests showed significance and the number of features that corresponded to each outcome. In addition, an archetype depicted in each Venn segment indicates the generalized structure of the feature-process relationships revealed by this statistical testing. Note that, while these archetypes illustrate where statistical differences do or do not arise, the actual sign of changes may also be inverted. Based on this overview, we can draw a variety of conclusions. First, we see that 92 of the 150 (61%) recorded features show some conditional dependence on Cell Speed. Interestingly, none of these features belong to the archetype defining an explicitly non-monotonic response (with significant differences observed for slow *versus* moderate, and for moderate *versus* fast, but not for slow *versus* fast). Nor are statistically significant non-monotonic responses to Cell Speed detected in the category where all three quintiles are distinct. Thus, although some features (noted below and in S2 Table) show weak non-monotonic trends, none are statistically significant and thus all feature relations to Cell Speed are approximately monotonic. Despite this, only 27 of the 92 features that reveal Cell Speed dependence show near-linear responses that are sensitive over the entire speed range (e.g. decreasing Mean [CMAC Lifetime] (Fig 4B) and decreasing Median [CMAC Mean paxillin] (Fig 4C)). An additional 8 features show changes only between slow and fast cells, suggesting a weak but again relatively linear response over the complete Cell Speed range (e.g. slowly decreasing QD [CMAC Compactness] (Fig 4D) and slowly decreasing Median [CMAC ΔArea] (Fig 4E)). In contrast, the remaining 65 features show non-linear, context-dependent relationships, suggesting that inter-feature dependencies evolve with changing migration speed. For example, 21 features are sensitive to changes between slow and moderate migration, yet remain constant between moderate and fast migration. Of these, 5 show no differences between slow and fast cells (hinting at weak non-monotonicity (e.g. increasing then slightly decreasing Cell major axis (Fig 4F)), while 16 features also differ between slow and fast cells, suggesting a plateauing relationship (e.g. initially increasing QD [CMAC to border distance] (Fig 4G) and initially decreasing Cell paxillin-LifeAct colocalization (Fig 4H)). A substantially greater number of features (36) are sensitive to changes between moderate and fast migration, but constant between slow and moderate migration speeds. Of these 36 features, 2 show no differences between slow and fast cells, again supporting weak non-monotonicity (e.g. slightly increasing then decreasing CoV [CMAC Speed] (Fig 4I)), while 34 also show differences between slow and fast cells, suggesting a plateauing response (e.g. slight then significant decreases in Number of CMACs (Fig 4J) and slight then significantly decreasing Cell Area (Fig 4K)).

### Feature relationships to Corrected Membrane Dynamics are relatively linear

We next applied an equivalent strategy to define the structure of relationships between features and Corrected Membrane Dynamics. Observations were stratified into quintiles based on CMD values, and pairwise comparisons of feature distributions were performed between quintiles 1 (low), 3 (intermediate), and 5 (high). Based on this combinatorial analysis, a Venn diagram (Fig 5A) reveals the frequencies at which relationships correspond with archetypal patterns. Overall, 75 of the 150 features tested (50%) show a correspondence with CMD, indicating a generally somewhat weaker relationship to Corrected Membrane Dynamics than that observed to Cell Speed (61%). As with Cell Speed, no explicitly non-monotonic correspondences with CMD were detected (with significant differences observed for low *versus* intermediate, and for intermediate *versus* high, but not for low *versus* high). Similarly, none of the features where quintiles 1, 3, and 5 were all distinct show evidence of non-monotonicity. In fact, the Venn diagram reveals that the majority of CMD-related features show approximately linear correspondences, with 28 features changing progressively at all CMD values (e.g. decreasing Mean [CMAC Lifetime] (Fig 5B) and decreasing Median [CMAC Mean LifeAct] (Fig 5C)), and 12 changing more slowly from low to high CMD values (e.g. decreasing CoV [CMAC Speed] (Fig 5D)). Thus, 40 of 75 CMD-related features (53%) show a linear relation, compared to just 35 of 92 Cell Speed-related features (38%). This suggests more direct (linear) relationships between Corrected Membrane Dynamics and underlying features, while more complex (non-linear, context-dependent) associations exist between these features and Cell Speed.

Despite a greater tendency towards linear relationships, non-linear, context-dependent relationships to CMD were also observed. Interestingly, as noted for quintiles 1 and 3 of Cell Speed, relatively few features (11) are sensitive to changes between low and intermediate CMD. Of these, 2 show no difference between low and high CMD (suggesting weak non-monotonicity, e.g. increasing then slightly decreasing QD [CMAC Angle] (Fig 5E)), while 9 features are distinct between low and high CMD (suggesting a plateauing response, e.g. initially increasing Number of CMACs (Fig 5F) and initially increasing Median [CMAC to center Distance] (Fig 5G)). This compares to 24 features that are sensitive to changes between intermediate and high CMD, of which 5 are insensitive to changes between low and high CMD (supporting weak non-monotonicity, e.g. slightly increasing then strongly decreasing Sum [CMAC paxillin Content] (Fig 5H) and slightly increasing then strongly decreasing Sum [CMAC LifeAct Content] (Fig 5I)). 19 features were also sensitive to differences between low and high CMD (indicative of a plateauing response, e.g. slowly decreasing Median [CMAC Mean paxillin] (Fig 5J) and slowly decreasing Median [CMAC Area] (Fig 5K)).

### Population-level dependencies are also observable in the dynamics of single cells over time

Using cell population data, we have mapped the presence and structure of relationships between quantitative cell, CMAC and F-actin features and Cell Speed or CMD. Yet it remains unclear whether these dependencies actually reflect dynamic variations that emerge as cells change their behavior over time or stable and confounding differences between cells in the sampled population. Therefore, we next assessed whether the statistical tendencies defined in our population data could also be observed within the time-series data from individual cells as their behavior changed over the 8 h imaging period. This required the identification of cells that transitioned between behavioral states (quintiles 1, 3, and 5 of Cell Speed or CMD) and occupied each state for sufficient time to allow a reasonable quantitative comparison of corresponding feature values (a rare occurrence). Importantly, Cell Speed-conditioned analyses of feature values from such as a cell (S2 Fig and S2 Movie) recapitulate many of the trends detailed by population data (Fig 4 and S1 Table). For example, as Cell Speed increased, we observed: an increase in cell length (S2C Fig–as in Fig 4F); increased spread of CMAC to border Distance (S2D Fig–as in Fig 4G); decreases in CMAC Lifetime (S2E Fig–as in Fig 4B); and decreased CMAC paxillin concentrations (S2F Fig–as in Fig 4C). Similar analysis of a representative cell whose trajectory traverses all CMD quintiles over time (S3 Fig and S3 Movie) mirrors observations derived from population data (Fig 5 and S1 Table). Heterogeneity over time in this single cell reveals that increased membrane dynamics correlate with: decreased CMAC Area (S3C Fig–as in Fig 5K); increased CMAC to center Distance (S3D Fig–as in Fig 5G); decreased CMAC Lifetime (S3E Fig–as in Fig 5B); and decreased CMAC paxillin concentration (S3F Fig–as in Fig 5J).

Taken together, these examples suggest that dependencies identified between underlying features and Cell Speed or CMD processes at the population-level also emerge within single cell trajectories. This supports the relevance of our population-based inferences to the dynamical regulation of individual cells over time.

### Disentangling features related to Cell Speed or Corrected Membrane Dynamics

**Fig 6:**
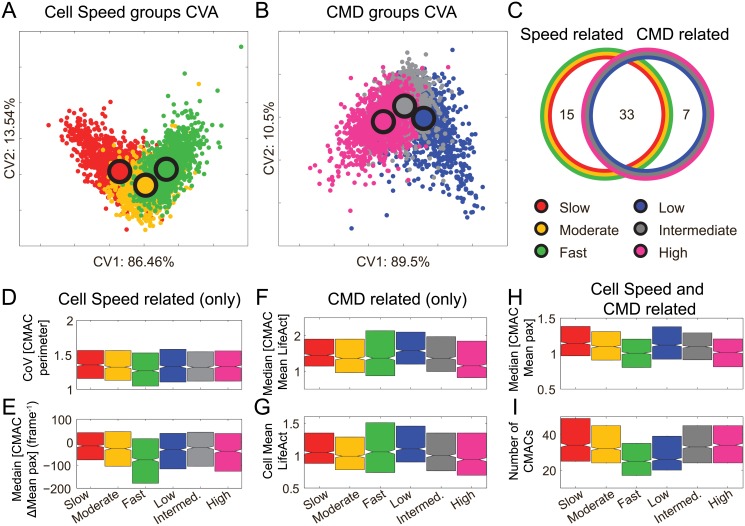
Stringent selection of features related to Cell Speed and or Corrected Membrane Dynamics. Canonical Vector Analysis (CVA) was used for multivariate separation of slow (red), moderate (yellow) and fast (green) Cell Speed groups (A), and low (blue), intermediate (gray) and high (pink) Corrected Membrane Dynamics (CMD) groups (B), respectively. (C) The features were categorized by whether they contributed to each separation, as well as whether they showed a significant difference between groups (determined via Kruskal-Wallis multiple group testing). According to this two-step criteria, 15 variables contributed to a difference only between Cell Speed related groups, 7 variables contributed to the difference between CMD related groups only and 33 variables contributed to both Cell Speed and CMD related differences. (D-E) Cell Speed related responses. (D) The coefficient of variation indicates heterogeneity in Cell-Matrix Adhesion Complex (CMAC) perimeter distribution. This heterogeneity decreases with Cell Speed but is not significantly changed with CMD. (E) The median rate of change in CMAC paxillin intensity (frame-to-frame difference) shows a concerted decrease in relation to increased Cell Speed, but not in response to changing CMD. (F-G) Responses related to CMD. (F) Median LifeAct intensity per CMAC. This feature is independent of Cell Speed, but decreases with higher CMD. (G) Mean LifeAct intensity per cell is also independent of Cell Speed but decreases with increased CMD. (H-I) Responses related to both Cell Speed and CMD. (H) Median of mean CMAC paxillin intensity per CMAC decreases with both increased Cell Speed and CMD. (I) Number of CMACs per cell decreases with increased Cell Speed but increases with CMD.

Having applied a sensitive statistical approach to define the structure (linear, non-linear; archetype form) of relationships between underlying quantitative features and two overlapping cell processes of interest (cell migration and membrane dynamics), we next statistically disentangled these associations to identify selective relationships. To achieve this, we used a stringent two-step approach to characterize features as related to global changes (rather than local, inter-quintile changes) in each process (see Materials and Methods). First, we applied multiple condition Kruskal-Wallis testing between three states (quintiles 1, 3 and 5) for each biological process, to identify significant feature variation at any level. Second, we applied an additional layer of verification using canonical vector analysis (CVA, Fig 6A and 6B). The latter multivariate approach helped to identify particular variables of significance from within groups of highly correlated (~ collinear) features. This is a key difference from the highly sensitive approach used to define relationship structures (as in Figs 4 and 5), since here we aimed to find only those features that strongly and uniquely related to the processes of interest. Accordingly, this rigorous approach defined three sets of features: Cell Speed-related; CMD-related, and; features related to both Cell Speed and CMD (Fig 6C).

We found that 15 features showed specificity in relation to differences in Cell Speed, and 7 features were preferentially related to membrane dynamics. 33 features were related to both of these biological processes (Fig 6C, S2 Table). The 15 features associated specifically with Cell Speed were predominantly related to variability in CMAC age, CMAC shape (Fig 6D) or rates of change in CMAC paxillin concentration (Fig 6E). Common characteristics of these features were that they indicate greater morphological homogeneity amongst CMACs and reduced dynamics in the concentrations of paxillin and F-actin at CMACs, as Cell Speed increases. In contrast, a majority of the seven features specifically related to CMD indicated changing F-actin status, including reduced F-actin concentrations at CMACs (Fig 6F) and overall lower F-actin concentrations per cell (Fig 6G) as CMD increased.

We also observed a number of features with approximately equivalent correspondences to both Cell Speed and CMD. For example, the concentration of paxillin in CMACs decreased as either migration or CMD increased (Fig 6H). Similarly, CMAC lifetimes became shorter as either Cell Speed or CMD increased (Figs 4B and 5B). The spatial spread of CMAC localizations, relative to the cell center or cell border, became more heterogeneous (greater) with increased Cell Speed or CMD (Figs 4G and 5G, respectively). Strikingly, in contrast to these examples where features responded equivalently to changes in either process, we also identified features whose responses to changing Cell Speed and CMD were either opposite or distinct. These features included the number of CMACs per cell (Fig 6I) and both cell and CMAC area (S2 Table), both of which decreased with Cell Speed but increased with CMD. This highlights the partial independence between cell migration and membrane dynamics hypothesized at the outset of this study, and further confirms the capacity of our heterogeneity-based, statistical approach to disentangle relationships underlying each process.

## Discussion

In this study, we have employed quantitative single cell imaging in combination with rigorous statistical analyses to identify the structure and specificity of correspondences between core *features* of the cell migration system—focusing on adhesions and F-actin–and the overlapping *processes* of cell migration and cell membrane dynamics. Crucially, the capacity to differentiate between these interrelated processes was provided by calculation of a Cell Speed-independent measure of membrane dynamics, termed Corrected Membrane Dynamics (CMD). This unique approach has now revealed a variety of selective relationships between underlying cellular, adhesion and F-actin features and either migration or membrane dynamics, as well as features that correspond with both processes. Most notably, our analyses collectively highlight the broad preferential coupling of adhesion complex features with cell migration, and of F-actin status with membrane dynamics. Furthermore, by also performing a conditional analysis with sensitivity to multiple levels of migration or membrane dynamics, we detected frequent non-linearity in feature relationships to Cell Speed and CMD, implying the context-dependent coupling of each process with underlying features. Moreover, we revealed an unexpected speed-dependent imbalance between the relative sizes of protruding and retracting cell domains, as well as showing that protrusion and retraction domain sizes are independent of cell size. These unanticipated findings hint at generalizable trends that couple cell size, cell migration and membrane dynamics. Each of these findings, considered in detail below, contributes to a more precise and distinct understanding of the fundamental biological processes of cell migration and membrane dynamics, as well as their differential relationships to adhesion and F-actin machinery.

It is striking that, based solely on the information embedded in natural cellular heterogeneity, it is possible to discern patterns of preferential association, first between cell migration and adhesion complex features, and then between cell membrane dynamics and F-actin status. This suggests a surprising degree of modularity in the functional influence of adhesion and F-actin machineries. More generally, the partial independence of migration and membrane dynamics originally hypothesized is well supported by identification of features selectively correlated with one process, but not the other. Even more persuasive, however, are instances where features correlate with both processes but have distinct responses, as observed for measures related to cell size, adhesion size and the number of adhesions per cell. Yet, despite this evidence of partial independence, the strong overall connection between migration and membrane dynamics is emphasized by the large number of features that respond equivalently given corresponding variations in both processes. Particularly notable amongst these are features reflecting paxillin intensity (concentration) in adhesions. Given the close association between paxillin and molecular components that link adhesions and F-actin (e.g. vinculin) [21, 30–32], this may reflect the physical coupling of adhesions and F-actin, which in turn may strengthen the behavioral coupling of migration and membrane dynamics. Indeed, this fits with a general functional characterization where the F-actin cytoskeleton is responsible for powering the dynamic processes necessary for migration (protrusion, retraction), but that adhesion machinery ultimately limits and coordinates the translation of dynamics into migration [11, 25], such that the status of adhesion machinery more closely predicts the behavioral output of the migration system.

It is important to note that many of the relationships detected between underlying features and cellular processes correspond with previous observations [23, 33], and were observable both in cell population data and in data based on the changing status of individual cells over time (single cell time-series data). While the single cell time-series data is inherently noisy due to its low statistical power, it supports the premise that population-derived correspondences reflect dynamic regulatory mechanisms, rather than differences between stable (non-exchanging) cell sub-populations. More broadly, the recapitulation of population trends based on single cell time-series data indicates the potential for ergodicity in measured cell dynamical behaviors–wherein the variability and dependencies observed in a single cell over time recapitulates, to a significant degree, the variability and dependencies observed in cell population data at any individual time point. However, it was relatively rare to find individual cells that sampled the broad behavioral space defined by the entire cell population, and hence this proposition requires additional investigation in future. While the behavioral space sampled by individual cells might be increased by lengthening the observation period (beyond 8 hours as used here), it may be that ergodicity arises only over one or more cell cycles, such that population-equivalent variability develops within cell lineages [34], but less frequently within individual cells. Understanding such dynamics may play an important role in interpreting behavioral variability in physiological contexts, such as in relation to cancer cell metastasis.

In addition to identifying both specific and general associations between underlying cellular features and either cell migration or membrane dynamics, we also mapped the structure of these relationships–as summarized by inclusion in one of seven classes of response archetype. This was achieved by defining multiple activity levels (for Cell Speed and CMD) between which statistically significant differences could be assessed for each feature. We thereby defined non-linear response patterns that would have been invisible using a global correlative analysis of all Cell Speed or CMD values. This analysis builds upon previous indications of context-dependence in the functional relationships underlying cell migration [23], where molecular perturbations were shown to induce plasticity in patterns of Granger-causal influence. Remarkably, though the current analysis is based on correlative rather than causal relations, we now indicate that similar plasticity may emerge spontaneously in accordance with natural heterogeneity in cell behaviors, as also indicated during the process of neutrophil polarization [35].

While we have focused heavily on aggregate membrane dynamics, i.e. the combination of both protrusive and retractive activity, it is also informative to note the trends that exist in the relative activity levels of these sub-processes. Indeed, when considering the frequencies of cell speed occurrences and rates of cell area changes, we find that slow cell movement has a high frequency and is associated with slight area growth, while fast cell movement is rare but is linked to rapid cell shrinking. This may correspond to indications that random migration speed is largely determined by cell rear retraction events, which are intrinsic to the process of cell symmetry breaking [36, 37]. Our data now suggest that the observed cell area distribution is a consequence of the negative correlation between the frequency of cell speed occurrences and the magnitude of corresponding cell area changes (frequent small area increases balance occasional large area decreases). Moreover, this surprising correlation, between cell movement and area changes, indicates that to maintain cell area values in a given range, motile behaviors must be transient, particularly in the case of rapid migration where associated reductions in area are large. Confirmation of this inference by autocorrelation-based analysis of cell speed (revealing lower speed persistence in fast cells) serves to further support the existence of a strong coupling between cell migration speed and both the sign and rate of cell area changes. Furthermore, the balance of these processes appears to be fine tuned by the frequency and temporal stability of dynamics. It is interesting to note that while we here indicate the anti-correlation of cell speed and *cell speed persistence* (i.e. cell speed autocorrelation), cell speed has recently been shown to positively correlate with *cell directional persistence* (i.e. the time for which a cell moves in a particular direction [38]). This implies that the persistence of cell speed and direction may be negatively correlated, suggesting counter-balanced regulation of speed and direction in cells. Further understanding the mechanisms defining these complex relationships is now an intriguing area for further exploration.

Another surprising observation is the fact that total (uncorrected) dynamic area in cells (i.e. protrusive domains, such as lamellipodia and filopodia, and retractive domains) is independent of cell size. This suggests that the activity of machinery dedicated to production of these dynamic cellular domains is relatively consistent, and is neither deterministic of, nor determined by, cell size. In protrusions this means, for example, stable activity of F-actin nucleation, polymerization and severing machinery [39], and in retractions, similar consistency in the activity of contractile machinery such as non-muscle myosin IIb [40]. Such consistency may arise from dependence of these activities on relatively stable machinery concentrations, which may be somewhat independent of cell shape and size.

Finally, it is also intriguing to consider whether Corrected Membrane Dynamics, defined here as a speed-corrected measure of absolute membrane dynamics, may actually represent a meaningful measure of cell migration efficiency. Essentially, since membrane dynamics and Cell Speed are linearly correlated, Corrected Membrane Dynamics provides a method to compare the relative levels of membrane dynamics associated with cell movement at a given speed. Hence, cells with low CMD values move with high efficiency, since they minimize the displacement of membranes necessary to achieve a particular rate of migration. Whether some cells are persistently more or less efficient by this measure remains to be seen, as does the more exciting prospect that such efficiency may represent a basis for selection. Such a possibility is at least conceivable in contexts where cell resources are limited, as, for example, may be the case during early metastatic egress from poorly vascularized tumors. Although in such settings protrusion and retraction dynamics may represent a necessary mechanism for sampling the three-dimensional environment, minimizing the energy required for such sensing would nonetheless represent a form of efficiency. Thus, when considering the invasive potential of cancer cells, for example, measures of migratory efficiency—as exemplified by Corrected Membrane Dynamics—may provide novel insights beyond those accessed through canonical measures focused purely on cell migration/invasion speed or even directionality.

In summary, this study was motivated by indications that cell migration and membrane dynamics in 2D have significantly different predictive power in relation to 3D cancer cell invasion [8], because this implies that the 2D processes reflect mechanisms with significant independence. We have here implemented a statistical approach to disentangle these processes based on natural heterogeneity in both underlying machinery-organization as well as in migration and membrane dynamics, recorded simultaneously on a per cell basis. This perturbation-independent approach was adopted because of the strong likelihood that even highly targeted disruption of either machinery would rapidly induce broad and confounding effects across both processes. Overall, we identified: a) features with selective correspondences to either Cell Speed or CMD; b) features with equivalent relationships to both processes, and; c) features with distinct correspondences to Cell Speed and CMD, as well as detailed structures for each relationship. While b) confirms the expected strong overlap between migration and membrane dynamics, both a) and particularly c) support the partial independence of these processes by highlighting precise areas of functional differentiation. This corroborates our initial biological hypothesis while also indicating the efficacy of the current statistical approach as a method to disentangle dependencies in highly integrated biological processes.

## Materials and Methods

### Cell culture

Cell culture was performed as described previously [23]. Briefly, H1299 (human non-small cell lung carcinoma, ATCC) cells were stably transfected with both EGFP-Paxillin and RubyRed-LifeAct. Double-expressing stable clones were maintained in RPMI 1640 medium (Gibco) supplemented with 1 mg/ml Geneticin (G-418 sulfate, Gibco), 1 mM Glutamine and 10% fetal bovine serum (Gibco). Before imaging, substrates were coated with 10 μg/ml of purified human Fibronectin at 37°C for 2 h and blocked with 1% heat denatured bovine serum albumin (Sigma-Aldrich) at 37°C for 1 h. 4000 cells per well were plated in fibronectin-coated 96-well glass bottomed plates (170 μm optical glass, Matrical Bioscience) and serum deprived for 24 h prior to imaging.

### Live cell confocal microscopy

Cells were imaged at 37°C with 5% CO_2_ and maintained in normal cell culture medium absent of serum. Confocal laser scanning microscopy was performed using a Nikon A1R microscope with a PlanApo VC 60X/1.4 numerical aperture oil immersion objective. 1024×1024 pixel images were recorded at 5 min time intervals for 8 h with a 0.21 μm/pixel resolution.

### Image analysis and feature extraction

Acquired images were analyzed using PAD software (version 6.3.13, Digital Cell Imaging Laboratories, Keerbergen, Belgium). Cell and CMAC segmentation was done based on EGFP-Paxillin intensity, identifying cell and CMAC boundaries. Tracking of these segmented features was performed over time, based on nearest neighbor analysis. Segmentation and tracking was validated by direct visual inspection of all images and sequences. We performed quantification of morphological and dynamic cell and CMAC features as described previously [23]. In particular Cell Speed was calculated as the distance between centers of area of consecutive frames divided by the time between frames (5 min). From every frame, 19 cell related features and 38 CMAC related features were extracted. These features were morphological, based on pixel intensities or related to cell or CMAC localization or dynamics.

In order to compare the cell and CMAC scales, statistical descriptions of the distribution of CMAC features were calculated for each cell at each time point (referred to as a CMAC cohort). These cohort descriptors measure the median, the quartile dispersion (QD), the quartile skewness (QS) and the coefficient of variation (CoV) of each feature of the CMACs per cell and point in time. To characterize the distribution of each feature in each CMAC cohort, we calculated the values of the first, second and third quartiles of these features; we defined *q*_*n*_ as the *n*th quartile value of each such feature. The median value is equal to *q*_2_. The other CMAC cohort descriptors were defined as:

$$\text{QD}=2\left(q_{3}\text{ - }q_{1}\right),$$

$$\text{QS}=\frac{q_{2}-\left(q_{3}-q_{1}\right)/2}{\text{QD}},$$

$$\text{CoV}=\text{QD}/q_{2}.$$

Thus, QD indicates the absolute variability of the cohort feature. QS indicates the asymmetry in a cohort feature distribution. CoV is the variability of a cohort feature standardized to its median value. In addition, we summed total CMAC Area, total EGFP-paxillin and LifeAct contents and calculated the mean CMAC Lifetime in each CMAC cohort. From the cell segmentation and CMAC cohorts, we extracted 150 features in each cell at each point in time. In total we observed 122 cells across 19 independent experimental repeats. This gave rise to 6419 cell observations, and 177938 CMAC observations.

### Calculation of Dynamic Cell Area

We compared consecutive, segmented cell images in order to identify protrusions and retractions using Matlab R2013b (The Mathworks, Natick, MA, USA). Protrusions were identified as regions present in a cell at a certain time point but absent in the previous. Retractions were defined as regions present at one time point but absent in the next time point. Short-lived regions are those regions that are present at only one time point but not in the ones consecutively before or after. This approach is similar to that applied by Veronika *et al*. [4] with the main difference that we used the actual position of cells instead of aligning the cell centers between frames. This reflects the actual motion of subcellular regions rather than comparing changes in cell shape over time and follows previously published methods [41, 42]. The Dynamic Cell Area is the sum of the area of protrusions, retractions and short-lived regions at a time point.

### Calculation of Corrected Membrane Dynamics

Dynamic Cell Area was plotted as a function of Cell Speed, with the density of observations also conditioned upon cell speed (conditional density) and represented by color-coding. Specifically, colors show the conditional density in form of the base 10 logarithm of a bivariate histogram where the total number of observations, in each Cell Speed bin, was normalized to 1 (Fig 3A). Conditional densities as shown in Figs 2D, 3D and S1 Fig were calculated in similar ways and conditioned on the binning along the horizontal axis. We performed a linear fit between Cell Speed and Dynamic Cell Area as:

Dynamic Cell Area=k*(Cell Speed)+m.

The parameters *k* and *m* where then used to correct for the dependence between Cell Speed and Dynamic Cell Area. We introduced Corrected Membrane Dynamics (CMD) as:

CMD=(Dynamic Cell Area) - k*(Cell Speed) - m,

CMD is thereby a way to quantify membrane dynamics independently of Cell Speed.

### Data parsing

At the first time point in a sequence, protrusions are undefined and at the last time point, retractions are undefined. In order to accurately measure the Dynamic Cell Area and CMD, the first and last frames of each sequence were excluded. The same applies to time points adjacent to occasional gaps in a sequence. These gaps occur at time points where the cell is not possible to segment due to loss of focus or when part of the cell is outside the recorded image frame.

### Data standardization

Data was standardized as previously published [23] with a few modifications. Briefly, the median intensities of the smallest CMACs (smallest 3 percentiles, corresponding to CMACs in the size range between 0.15 and 0.2 μm^2^) in each experimental repeat were calculated, and CMAC intensities in each experimental repeat were standardized to these median values.

Note that all data, reflecting the completion of each of the methodological steps described to this point, are included as S1 Dataset.

### Autocorrelation Analysis of Cell Speed

The autocorrelation analysis of Cell Speed was performed per cell trajectory in order to measure the decay in correlation of Cell Speed for each cell over several time lags. Missing Cell Speed values were replaced by the mean value of Cell Speed for that cell trajectory and the autocorrelation was calculated using the Matlab function xcov. The mean autocorrelation coefficient over time lag intervals of up to 12 frames (1 hour) were calculated and compared for cells with different mean Cell Speed.

### Analysis of feature-process relationship structures

The structure of relationships between individual features and either Cell Speed or CMD was assessed by first stratifying observations according to quintiles of either process. These were composed as follows: quintile 1 = 0%-20% (termed “slow” for Cell Speed, “low” for CMD); quintile 2 = 20%-40%; quintile 3 = 40%-60% (termed “moderate” for Cell Speed, “intermediate” for CMD); quintile 4 = 60%-80%; quintile 5 = 80%-100% (termed “fast” for Cell Speed, “high” for CMD). Pairwise comparison was then performed between feature value distributions belonging to the first, third and fifth quintiles of Cell Speed or CMD using the Wilcoxon rank sum test. This was done for our set of 150 cell, CMAC and F-actin features and each pair of the three quintiles. In total 3×150 = 450 comparisons were made. We consider a difference that gives rise to a P value below 0.001 significant, when modified via a Bonferroni correction for the 450 comparisons performed. Features showing at least one significant difference were categorized based on the combination of tests that were significant, per process (Cell Speed or CMD). This categorization is summarized via Venn diagrams, with a generalized archetypical response shown for each category.

### Feature comparison

Step 1. In order to compare features that contribute to either Cell Speed or CMD, a Kruskal-Wallis test was performed for each feature, between groups defined by quintiles 1, 3, and 5 of each process. A P value less than 0.001 indicates significance, given a Bonferroni correction for 150 comparisons (one test for each feature).

Step 2. To limit the number of linearly dependent features categorized as describing differences between the groups, canonical vector analysis (CVA) [43] of the same groups was also performed. The sum of squares of the CVA load for each feature was calculated along each canonical vector and only features belonging to the top half of the list of features sorted by this sum were taken into consideration as being associated with either Cell Speed or CMD.

The lists of features contributing significantly to Cell Speed or CMD (Step 1) were compared to the lists of features taken into consideration from CVA of each process (Step 2). Features passing criteria at steps 1 and 2 for either one or both processes were categorized as related to Cell Speed, CMD or both of these processes.

## Supporting Information

S1 Dataset Dataset described in the paper. The dataset contains three sheets describing the quantitative features and processes for each cell observation. The third sheet contains an index of the observations, Each sheet contains 6419 rows corresponding to the observations of each cell at each time point. The order of the observations is the same in each sheet. The features sheet contains the 150 features describing the state of the cells. The processes sheet contains corresponding quantification of the processes of interest (Cell Speed, numbers of Protrusion Pixels, Retraction Pixels and Short-lived Pixels as well as Dynamic Cell Area and Corrected Membrane Dynamics). The index sheet contains the experimental date, Cell Trace identifier, the locations of the cells at each time point and the Frame Number.
(XLSB)

S1 Fig No correlation is detectable between Cell Area and Dynamic Cell Area. (A) Cell Area is plotted against absolute Dynamic Cell Area. The density of observations at a given Cell Area (Cell Area conditional density) is color-coded following log transformation, enabling better observation of trends in Dynamic Cell Area values given changing Cell Area. (B) Cell Area is plotted against absolute Dynamic Cell Area divided by Cell Area. The density of observations at a given Cell Area (Cell Area conditional density) is color-coded following log transformation, enabling better observation of trends in Dynamic Cell Area. Black lines show linear fits between features. We did not detect any correlation between Dynamic Cell Area and total Cell Area (A), while Dynamic Cell Area as a proportion of total Cell Area is negatively correlated with total Cell Area (B). Pearson’s correlation coefficient is *r* = -0.40.
(PDF)

S2 Fig Difference between cell and CMAC properties in relation to Cell Speed in a single cell. (A) The quantitative trajectory of a single cell over time within a Cell Speed-CMD plot. This cell traverses much of the Cell Speed range sampled by the total cell population data. Trajectory color-coded by time as indicated to the right. (B) Sample images show the morphology of the Cell, CMACs and F-actin at time points in the trajectory occupying quintiles 1 (slow, frame 13), 3 (moderate, frame 40) or 5 (fast, frame 57) of Cell Speed, thus illustrating the changes that accompany altered behavior. Images show EGFP-paxillin (green) and RubyRed-LifeAct (red) expression. Scale bar: 10 μm. See also S2 Movie. (C-F) Box plots showing feature value changes between Cell Speed quintiles (1, slow, red; 3, moderate, yellow; 5, fast, green) for the single cell detailed in (A): (C) Cell major Axis, (D) spread of Cell-Matrix Adhesion Complex (CMAC) to border distance, (E) Mean CMAC Lifetime per cell observation and (F) Median paxillin concentration in CMACs are shown. Boxes show quartiles. Whiskers show either maximum and minimum values or 1.5 times the interquartile range, whichever is closer to the median value of each feature. Outliers are not displayed.
(PDF)

S3 Fig Difference between cell and CMAC properties in relation to Corrected Membrane Dynamics in a single cell. (A) The quantitative trajectory of a single cell over time within a Cell Speed-CMD plot. This cell traverses much of the CMD range sampled by the total cell population data. Trajectory color-coded by time as indicated to the right. (B) Sample images from time points associated with quintiles 1 (low, frame 12), 3 (intermediate, frame 40) and 5 (high, frame 89) CMD depict the morphological alterations that correspond with changing behavior. Images show EGFP-paxillin (green) and RubyRed-LifeAct (red) expression. Scale bar: 10 μm. See also S3 Movie. (C-F) Box plots showing feature value changes between CMD quintiles (1, low, blue; 3, intermediate, grey; 5, high, pink) for the single cell detailed in (A): (C) the Median Cell-Matrix Adhesion Complex (CMAC) area, (D) Median CMAC to center distance, (E) Mean CMAC lifetime and (F) Median paxillin concentration in CMACs are shown. Boxes show quartiles. Whiskers show either maximum and minimum values or 1.5 times the interquartile range; whichever is closer to the median value of each feature. Outliers are not displayed.
(PDF)

S1 Movie Live cell imaging and identification of protrusions, retractions and short-lived regions. Left: A representative H1299 cell expressing EGFP-paxillin (green) and RubyRed-LifeAct (red), imaged at 5 min intervals. Right: The same cell is shown after segmentation and tracking. Protrusions (green), retractions (red), short-lived (blue) and stable (gray) regions were identified in each frame. The locations of Cell-Matrix Adhesion Complexes (CMACs) are indicated by white circles. Scale bar: 10 μm. See also Fig 2.
(MP4)

S2 Movie Trajectory of a single cell with a large change in speed. An example of a migrating cell with a large change in Cell Speed is shown. This is the same cell as in S2A Fig. Left: EGFP-paxillin (green) and RubyRed-LifeAct (red), imaged at 5 min intervals. Scale bar: 10 μm. The trajectory shows the motion of the centroid of the cell. Right: The trajectory through the Cell Speed-Corrected Membrane Dynamics plane is shown for the same cell.
(MP4)

S3 Movie Trajectory of a single cell with a large change in Corrected Membrane Dynamics. An example of a migrating cell with a large change in Corrected Membrane Dynamics (CMD) is shown. This is the same cell as in S3A Fig. Left: EGFP-paxillin (green) and RubyRed-LifeAct (red), imaged at 5 min intervals. Scale bar: 10 μm. The trajectory shows the motion of the centroid of the cell. Right: The trajectory through the Cell Speed-CMD plane is shown for the same cell.
(MP4)

S1 Table List of all features and their relationships to Cell Speed and/or Corrected Membrane Dynamics. The table lists the 150 features included in the analysis. The spatial scale indicates whether a measured feature is derived at the cellular scale or is based on the Cell-Matrix Adhesion Complex (CMAC) cohort, i.e. a statistical measure of the CMAC distribution per cell at a given time point. The feature class indicates which aspect of cell or CMAC properties is measured by a particular feature. The name of each feature specifies the exact quantity and, where relevant, the type of cohort statistics that it reflects (see Materials and Methods). For each feature the unit is specified. The six “dependence” columns indicate where significant differences where observed between pairs Cell Speed or CMD groups stratified as quintiles and tested pairwise as in Figs 4 and 5. Columns E-G (light green background) summarize feature relationships to Cell Speed. Columns H-J (light blue background) summarize feature relations to CMD. Up (red) indicates an increase with either process, Down (blue) a decrease and ns indicates no significance. The last column contains a brief description of each feature.
(XLSX)

S2 Table Lists of features related to Cell Speed, Corrected Membrane Dynamics or both processes. The table lists features categorized by whether they are related to Cell Speed, Corrected Membrane Dynamics (CMD) or both of these biological processes. The type of association is also listed as: Increase (red); Decrease (blue); Min (yellow, meaning that the middle state has the lowest value), or; Max (green, meaning that the middle state has the highest value) for that particular feature. Within each type of relation between a feature and a process, features are grouped by their type of association with a broader description of the cell or its Cell-Matrix Adhesion Complexes (CMACs).
(XLSX)
