# Supplementary figures and images for: Disentangling Membrane Dynamics and Cell Migration; Differential Influences of F-actin and Cell-Matrix Adhesions
Source: PLoS One. 2015 Aug 6;10(8):e0135204. doi: 10.1371/journal.pone.0135204 (PMC4527765; doi:10.1371/journal.pone.0135204)

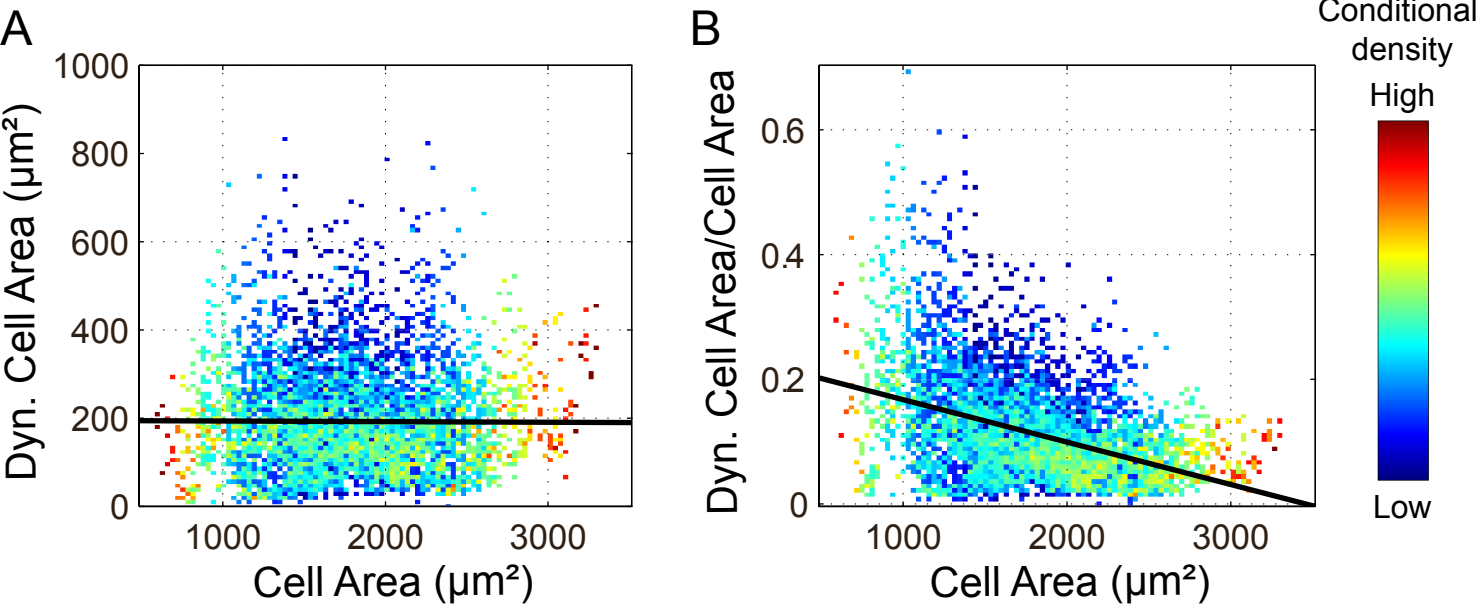

S1 Figure\_Kowalewski et al

Supplement: S1 Fig — (A) Cell Area is plotted against absolute Dynamic Cell Area. The density of observations at a given Cell Area (Cell Area conditional density) is color-coded following log transformation, enabling better observation of trends in Dynamic Cell Area values given changing Cell Area. (B) Cell Area is plotted against absolute Dynamic Cell Area divided by Cell Area. The density of observations at a given Cell Area (Cell Area conditional density) is color-coded following log transformation, enabling better observation of trends in Dynamic Cell Area. Black lines show linear fits between features. We did not detect any correlation between Dynamic Cell Area and total Cell Area (A), while Dynamic Cell Area as a proportion of total Cell Area is negatively correlated with total Cell Area (B). Pearson’s correlation coefficient is r = -0.40. (PDF) [file pone.0135204.s002.pdf]

S2 Figure\_Kowalewski et al

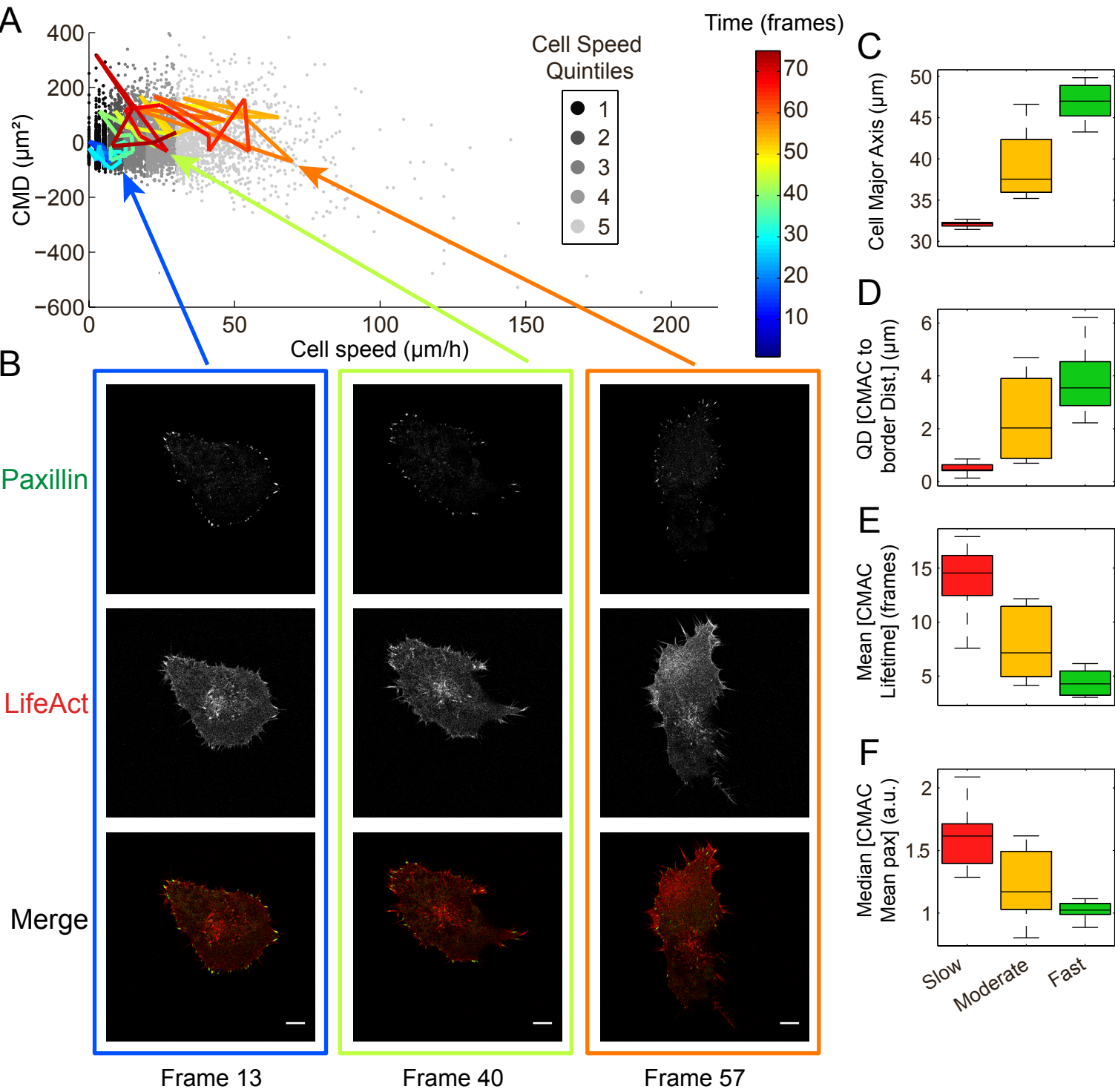

Supplement: S2 Fig — (A) The quantitative trajectory of a single cell over time within a Cell Speed-CMD plot. This cell traverses much of the Cell Speed range sampled by the total cell population data. Trajectory color-coded by time as indicated to the right. (B) Sample images show the morphology of the Cell, CMACs and F-actin at time points in the trajectory occupying quintiles 1 (slow, frame 13), 3 (moderate, frame 40) or 5 (fast, frame 57) of Cell Speed, thus illustrating the changes that accompany altered behavior. Images show EGFP-paxillin (green) and RubyRed-LifeAct (red) expression. Scale bar: 10 μm. See also S2 Movie. (C-F) Box plots showing feature value changes between Cell Speed quintiles (1, slow, red; 3, moderate, yellow; 5, fast, green) for the single cell detailed in (A): (C) Cell major Axis, (D) spread of Cell-Matrix Adhesion Complex (CMAC) to border distance, (E) Mean CMAC Lifetime per cell observation and (F) Median paxillin concentration in CMACs are shown. Boxes show quartiles. Whiskers show either maximum and minimum values or 1.5 times the interquartile range, whichever is closer to the median value of each feature. Outliers are not displayed. (PDF) [file pone.0135204.s003.pdf]

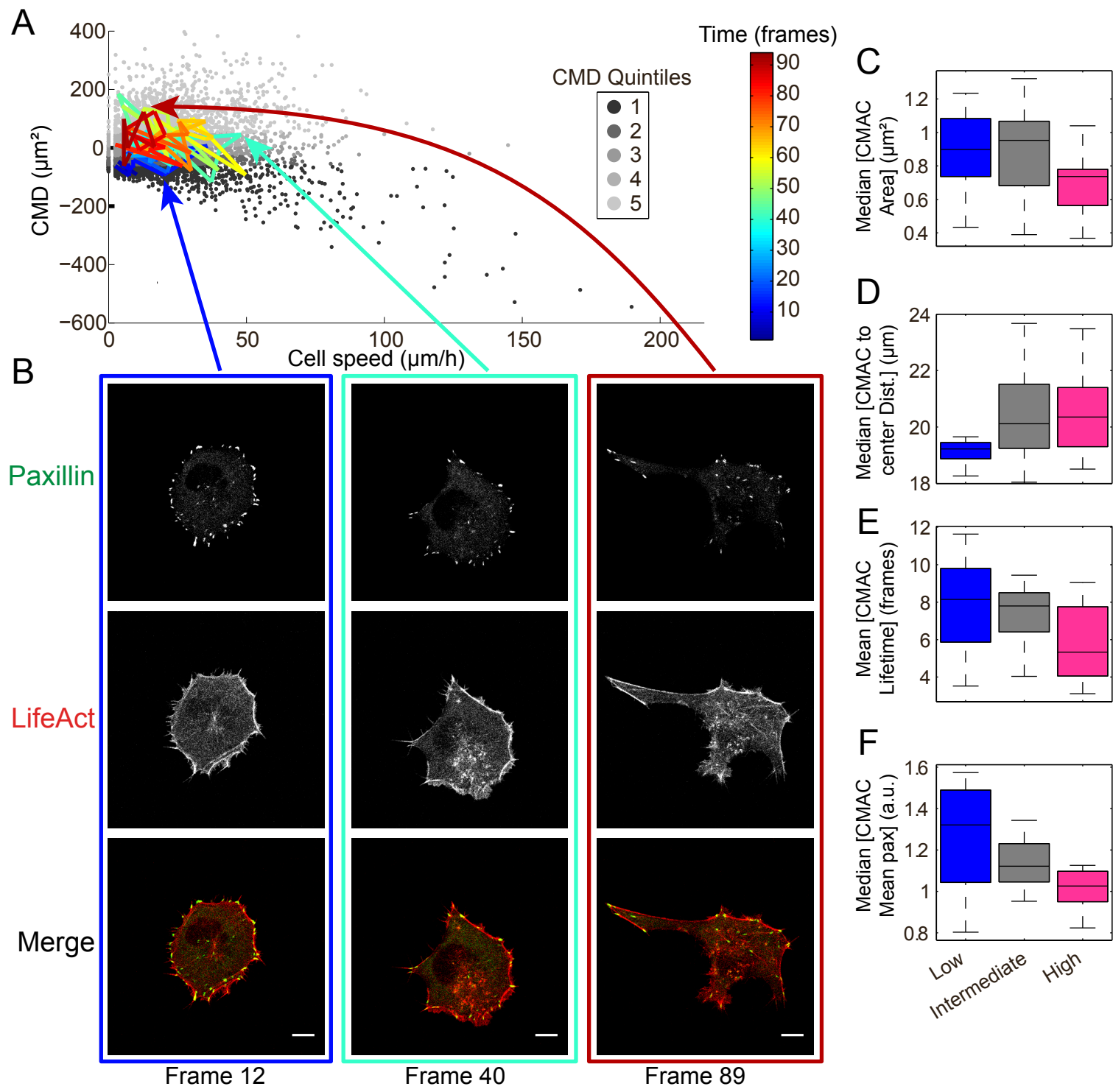

Supplement: S3 Fig — (A) The quantitative trajectory of a single cell over time within a Cell Speed-CMD plot. This cell traverses much of the CMD range sampled by the total cell population data. Trajectory color-coded by time as indicated to the right. (B) Sample images from time points associated with quintiles 1 (low, frame 12), 3 (intermediate, frame 40) and 5 (high, frame 89) CMD depict the morphological alterations that correspond with changing behavior. Images show EGFP-paxillin (green) and RubyRed-LifeAct (red) expression. Scale bar: 10 μm. See also S3 Movie. (C-F) Box plots showing feature value changes between CMD quintiles (1, low, blue; 3, intermediate, grey; 5, high, pink) for the single cell detailed in (A): (C) the Median Cell-Matrix Adhesion Complex (CMAC) area, (D) Median CMAC to center distance, (E) Mean CMAC lifetime and (F) Median paxillin concentration in CMACs are shown. Boxes show quartiles. Whiskers show either maximum and minimum values or 1.5 times the interquartile range; whichever is closer to the median value of each feature. Outliers are not displayed. (PDF) [file pone.0135204.s004.pdf]
